# Aliphatic hydrocarbons in fin spines of adult sturgeon (*Acipenser stellatus*) and their relationship with potentially toxic elements in the northern and southern regions of the Caspian Sea

**DOI:** 10.1007/s11356-024-32653-y

**Published:** 2024-03-01

**Authors:** Shima Bakhshalizadeh, Botagoz Nasibulina, Tatyana Kurochkina, Attaala Ali, Rafael Mora-Medina, Nahúm Ayala-Soldado

**Affiliations:** 1 Department of Marine Science, Caspian Sea Basin Research Center, University of Guilan, Rasht, Iran; 2 Faculty of Geology & Geography, Innovative Natural Institute, Astrakhan State University, Astrakhan, Russia; 3 Hadhramout University, Marine Biology, Mukalla, Yemen; 4 Department of Anatomy and Comparative Pathology and Toxicology, Faculty of Veterinary Medicine, University of Córdoba, Cordoba, Spain

**Keywords:** Arsenic, Bioaccumulation, Endangered fish, Heavy metals, Petroleum hydrocarbons

## Abstract

Currently, the pollution of the Caspian Sea by the oil industry is one of the highest problems in this area. Critically endangered species inhabit this sea, such as sturgeons, whose ecological value is incalculable. Thus, we aimed to evaluate the level of contamination of aliphatic hydrocarbons of petroleum and its relation with several toxic elements directly on sturgeons spines. A total of 40 adult starry sturgeons (*Acipenser stellatus*) were obtained within a repopulation programme in the northern and southern coastal waters of the Caspian Sea. The marginal pectoral fin was extracted from each fish to determine aliphatic hydrocarbons, arsenic, cadmium, mercury, nickel, lead, and vanadium. Subsequently, the sturgeons were released. Clearly, the presence of hydrocarbons was evidenced in all the sampled areas finding higher concentrations in the northern areas (N1 = 1.35 ± 0.4; N2 = 1.65 ± 0.46; N3 = 1.27 ± 0.40; S1 = 0.61 ± 0.22; S2 = 0.85 ± 0.43 mg/kg). Furthermore, to a greater or lesser extent, some toxic elements, mainly Hg and As, have been linked to aliphatic hydrocarbons.

## Introduction

The Caspian Sea is the largest enclosed body of water in the world, with a surface of approximately 370,000 km^2^ and a volume of water of 78,000 km^3^. It is located between Asia and Europe and is bordered by five countries: Russia, Kazajistan, Turkmenistan, Iran, and Azerbaiyan. Based on its geophysical conditions, this sea can be subdivided into three parts: the northern area with a maximum depth of 30 m and a salinity of below 10 g/L, and the middle and southern areas, with a salinity of nearly 13 g/L and depths of 800 and 1000 m, respectively (Kosarev 2005; Lattuada et al. 2019). It is filled and drained mainly by the rivers Volga (233 km^3^/year) and Ural (6.6 km^3^/year), as well as the rainfall (Ramazanova et al. 2022). The fact that it is closed makes its ecosystem less tolerant and more vulnerable to human-generated and spilled environmental pollutants (Ghayebzadeh et al. 2020).

Currently, pollution from crude oil is one of the most important concerns in the Caspian Sea. The first deep wells began to be excavated in the nineteenth century and, from that moment on, both the extensive exploitation of the oil and its transport across the Volga river have originated drastic pollution in this area. During the Soviet period, the oil and gas companies were principally limited to the west and north coasts. After its dissolution, all the countries on its coasts have become involved in this industry (Leroy et al. 2020). Crude oil is classified in four well-defined fractions: aliphatic, aromatic hydrocarbons, resins, and asphaltenes. Aliphatic hydrocarbons represent the greater part of crude oil, and they have no double bonds (Varjani 2017; Oyibo et al. 2018). In addition, the oil may contain a variety of potentially toxic elements like arsenic (As), cadmium (Cd), lead (Pb), nickel (Ni), mercury (Hg), and vanadium (V), which mainly originate in crude oil from the perforation fluid during its extraction, and in the detachment of impurities from the oil products and tanks (Wilhelm and Bloom 2000; Fakhru’l-Razi et al. 2009; Li et al. 2021). To be specific, the most abundant metals in crude oil and its by-products are Ni and V. Both metals are joined in the centre of some aromatic compounds called petroporphyrins, forming V or Ni petroporphyrins (Valencia 2023).

In the Caspian Sea, the total ichthyofauna reaches 76 species and 47 subspecies, in reference to 17 families and 53 genera (Leroy et al. 2020). Among these 76 species, there are five species of sturgeon, including Persian sturgeon (*Acipenser persicus*), Russian sturgeon (*Acipenser gueldenstaedti*), starry sturgeon (*Acipenser stellatus*), boat sturgeon (*Acipenser nudiventris*), and beluga sturgeon (*Huso huso*) (Wang et al. 2008). Although sturgeon caviar is currently produced in farms (Bronzi et al. 2019), in the past, approximately 90% of sturgeon caviar comes from this sea (Pourkazemi 2006; Graham and Murphy 2007). However, overfishing and the contamination of the ecosystem have greatly affected sturgeon populations. Actually, in the Caspian Sea, these five sturgeon species have been classified as being “critically endangered species” by the International Union for the Conservation of Nature (UICN 2022). Thus, there is an urgent need for the five surrounding countries to develop a common strategy for the revival of sturgeon populations (FAO 2022).

For all the above, our study aimed to determine the concentrations of total aliphatic hydrocarbons (TAH) and the potentially toxic elements, As, Cd, Hg, Pb, V, and Ni in the pectoral fin of the starry sturgeon (*A. stellatus*) in different places of the northern and southern areas of the Caspian Sea. We set two main objectives: (i) to evaluate the differences in the concentrations of these elements in the different zones and (ii) to establish the relationships between concentrations of hydrocarbons and toxic elements.

## Material and methods

### Sampling area and preparation of the samples

**Fig. 1:**
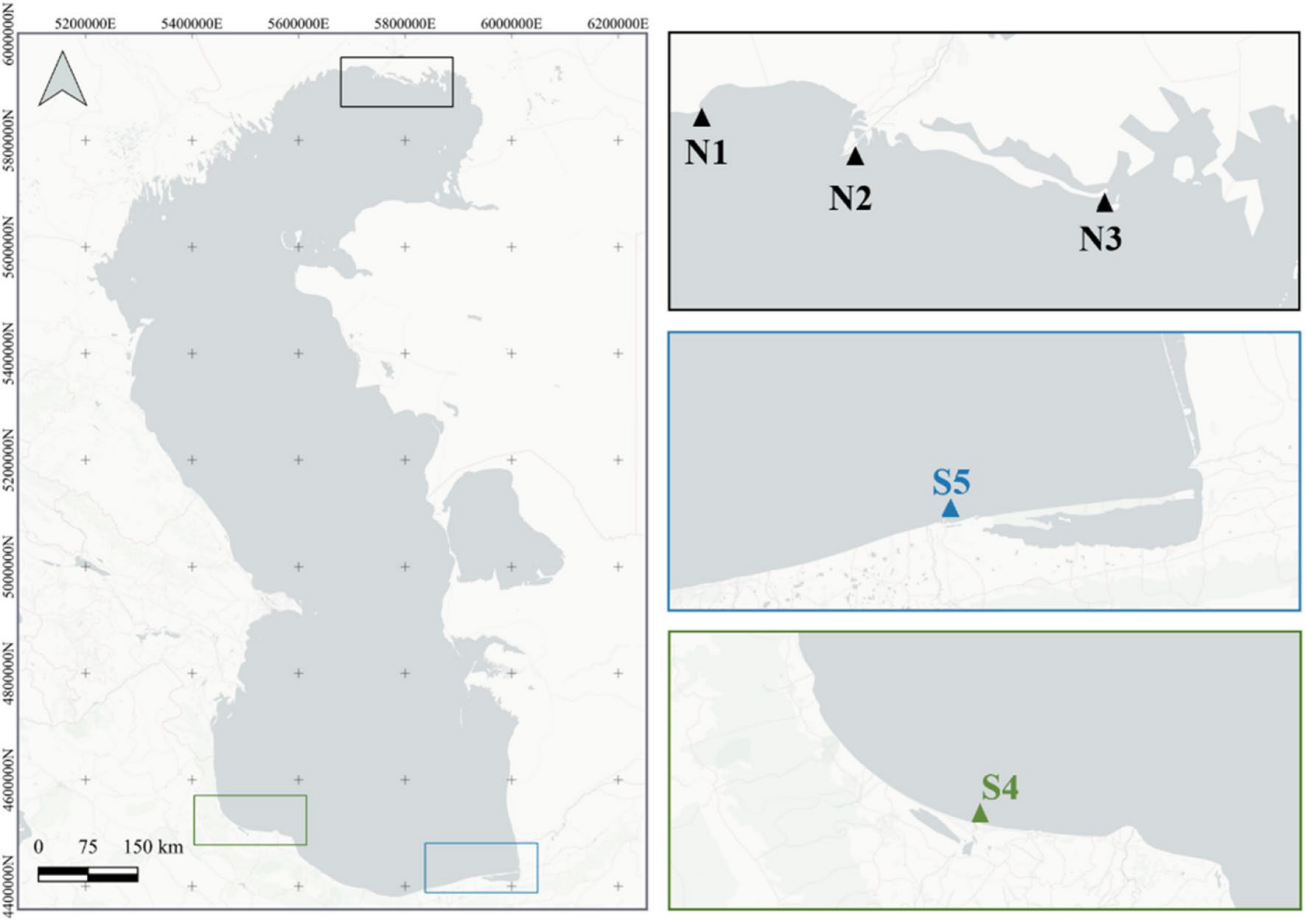
Different areas in which starry sturgeons are caught in the Caspian Sea

A total of 40 adult starry sturgeons with a mean weight of 6.38 ± 1.55 kg and 134.57 ± 13.57 cm long were obtained within a repopulation programme in the northern and southern coastal waters of the Caspian Sea. Figure 1 shows sampling sites. More precisely, three sampling areas were established in the north (N1, N2, and N3), in which a total of 20 specimens were analyzed (eight, four, and eight, respectively), and two in the south (S4 and S5), where another 20 specimens were evaluated, 10 per sampling area.

The marginal pectoral fin ray (the most cranial one) was extracted from each fish by cutting it at the articulation point, following Koch et al. (2008). Before taking each sample, the instruments used were rinsed in 1% nitric acid. The samples were frozen at − 80 °C up to analysis. It is important to clarify that this sampling method is not lethal. Fish were not sacrificed for this study but were freed once the samples had been taken. This work was approved by the Ethics Committee of the institution involved, and was carried out in accordance with the European regulations for the protection of animals used for scientific purposes (European Parliament 2010).

### Potentially toxic element analyses

The spine samples were digested with a mixture of nitric acid (65% HNO_3_) and perchloric acid (60% HClO_4_) (2:1 v/v) in a boiling water bath at 70 °C to decompose all the organic matter. The solution obtained was completed with distilled water until reaching a volume of 50 ml. The toxic element concentrations were quantified by employing an inductively coupled plasma-mass spectrometer (ICP-MS) with Agilent 7500 series equipment. Analytical blanks were processed appropriately, and concentrations were determined using standard solutions that were prepared in the same acidic matrix.

### TAH analyses

The concentration of the following aliphatic hydrocarbons was determined: C10, C11, C12, C13, C14, C15, C16, C17, Pristane, C18, Phytane, C19, C20, C21, C22, C23, C24, C25, C26, C27, C28, C29, C30, C31, C32, C33, C34, C35, C36, C37, C38, and C39. After the individual determinations, their concentrations were summed to obtain the TAH value for each sturgeon.

For the extraction, each sample was ground in a clean mortar together with 10 g of anhydrous sodium sulfate until completely dry and homogenized. The sample was extracted using 50 ml of hexane with dichloromethane employed as the extraction solvent in a 1:1 ratio. Ten grams of the homogenized product of the sample was placed in a 50-ml extraction bottle, which was left for 60 min in an ultrasonic bath. The extract was carefully decanted and concentrated to 2 ml using a rotary evaporator kept at 20 °C. The aliphatic hydrocarbons concentrations were determined by means of gas chromatography with Agilent-7890 equipped with a mass spectrometer (Agilent-5975) with a quadrupole type Split-Splitless inlet.

### Statistical analysis

Statistical analysis of the data was performed using the SPSS version 25 statistical software. The normality of the data was assessed using the Kolmogorov–Smirnov test. Due to the non-normality of the TAH and toxic element concentrations, the data were analyzed by the Kruskal-Walis non-parametric *H* test to compare the concentrations between zones. Bonferroni correction was used as a method of multiple comparisons in pairwise analysis. It is important to highlight that Bonferroni correction as a pairwise comparison is a much more conservative method than other statistical analyses (Chen et al. 2017), thus ensuring that the differences found are representative. Besides, to evaluate the differences between north and south in general (∑N1-N3 and ∑S1-S2), the non-parametric Mann–Whitney *U* test was used.

On the other hand, to establish the relationships between elements, Spearman’s correlation test was applied. In all the cases, a value of *p* < 0.05 was taken as being significant. Finally, to study the relationships of the elements with hydrocarbons, a principal component analysis (PCA) was carried out in R studio using the *FactoMiner* and *Factoextra* libraries. The length and weight data of the animals were included in this analysis.

## Results

### TAH and toxic element concentrations

The descriptive statistics of the TAH and toxic element concentrations in the different sampling areas of the Caspian Sea are shown in Table 1. Regarding TAH concentrations, significantly higher concentrations were found in zone N1 compared to zone S4. For As, zones N1 and N2 had significantly higher concentrations than zone S5. In the case of Pb and Cd, no differences attributable to location were found. Ni levels were substantially higher in zones N1 and N3 than in zone S5. Finally, V concentrations were higher in N1 than N3 and S5 zones. In general, except for Pb and Cd, some northern location always had a higher concentration of TAH and toxic elements than some southern zone. In fact, on analyzing the samples from the northern and southern areas together, i.e., ∑N1–N3 and ∑S4–S5, it was found that the northern area presented significantly higher concentrations (*p* < 0.05) of TAH, As, Hg, Ni, and V. Specifically, mean TAH levels in north were 1.380 ± 0.416 mg/kg, while in the south, they were significantly lower, 0.737 ± 0.356 mg/kg. Regarding toxic elements, As levels were 1.943 ± 2.805 µg/kg in north compared to 0.291 ± 0.601 µg/kg in south. North Hg concentrations were 1.076 ± 0.800 µg/kg compered to south Hg levels of 0.520 ± 1.576 µg/kg. Ni concentrations in south were 1.314 ± 1.731 µg/kg compered to higher concentrations in north (3.995 ± 3.837 µg/kg). Finally, V levels were 13.306 ± 5.475 and 1.314 ± 1.731 µg/kg in the north and south of the Caspian Sea, respectively. An interesting fact obtained in this study is that several sturgeons from southern areas had levels of As, Hg, and Cd lower than that of the detection limit of the test.

**Table 1:** Toxic element and TAH concentrations in starry sturgeons in different areas of the Caspian Sea

Zone		Parameter	*n*	Mean	SD	Median	Min	Max
North	N1	TAH (mg/kg)^a^	8	1.351	0.402	1.437	0.784	2.015
		As (µg/kg)^a^		3.248	3.753	1.487	0.000	8.804
		Cd (µg/kg)		0.268	0.152	0.214	0.077	0.506
		Hg (µg/kg)^a^		1.502	1.117	1.031	0.447	3.676
		Ni (µg/kg)^a^		5.732	4.650	3.956	0.000	13.501
		Pb (µg/kg)		10.485	4.912	8.065	7.084	21.441
		V (µg/kg)^a^		17.782	6.214	15.590	14.269	32.952
	N2	TAH (mg/kg)^a^	4	1.651	0.458	1.719	1.030	2.134
		As (µg/kg)^a^		1.994	2.597	0.710	0.667	5.890
		Cd (µg/kg)		0.191	0.082	0.194	0.090	0.285
		Hg (µg/kg)^a,b^		0.856	0.146	0.812	0.741	1.059
		Ni (µg/kg)^a,b^		2.243	1.608	1.911	0.695	4.456
		Pb (µg/kg)		11.474	4.911	10.253	7.165	18.226
		V (µg/kg)^a,b^		12.134	0.642	12.239	11.263	12.795
	N3	TAH (mg/kg)^a^	8	1.273	0.403	1.315	0.411	1.796
		As (µg/kg)^a,b^		0.611	0.635	0.473	0.000	1.615
		Cd (µg/kg)		0.177	0.031	0.181	0.116	0.207
		Hg (µg/kg)^a,b^		0.761	0.364	0.640	0.373	1.472
		Ni (µg/kg)^a^		3.134	3.342	2.377	0.000	9.470
		Pb (µg/kg)		8.078	2.864	6.963	5.957	14.740
		V (µg/kg)^b^		9.415	1.259	9.494	7.195	10.763
South	S4	TAH (mg/kg)^b^	10	0.609	0.215	0.578	0.427	1.122
		As (µg/kg)^a,b^		0.404	0.716	0.115	0.000	2.319
		Cd (µg/kg)		0.270	0.269	0.188	0.064	1.001
		Hg (µg/kg)^b^		0.817	2.180	0.112	0.000	7.013
		Ni (µg/kg)ab		2.245	1.713	1.871	0.000	4.474
		Pb (µg/kg)		8.767	3.446	7.669	5.284	16.551
		V (µg/kg)^a,b^		14.497	11.237	10.170	7.186	44.076
	S5	TAH (mg/kg)^a,b^	10	0.851	0.427	0.790	0.143	1.759
		As (µg/kg)^b^		0.178	0.494	0.000	0.000	1.578
		Cd (µg/kg)		0.126	0.059	0.103	0.064	0.246
		Hg (µg/kg)^b^		0.224	0.545	0.000	0.000	1.758
		Ni (µg/kg)^b^		0.382	1.210	0.000	0.000	3.827
		Pb (µg/kg)		7.919	3.110	7.529	4.096	15.838
		V (µg/kg)^b^		6.390	3.568	5.203	2.391	13.049

^a,b ^When at least one letter is not shared in the same compound, there are statistically significant differences (*p* < 0.05)

### Correlation analysis between TAH and toxic elements

**Fig. 2:**
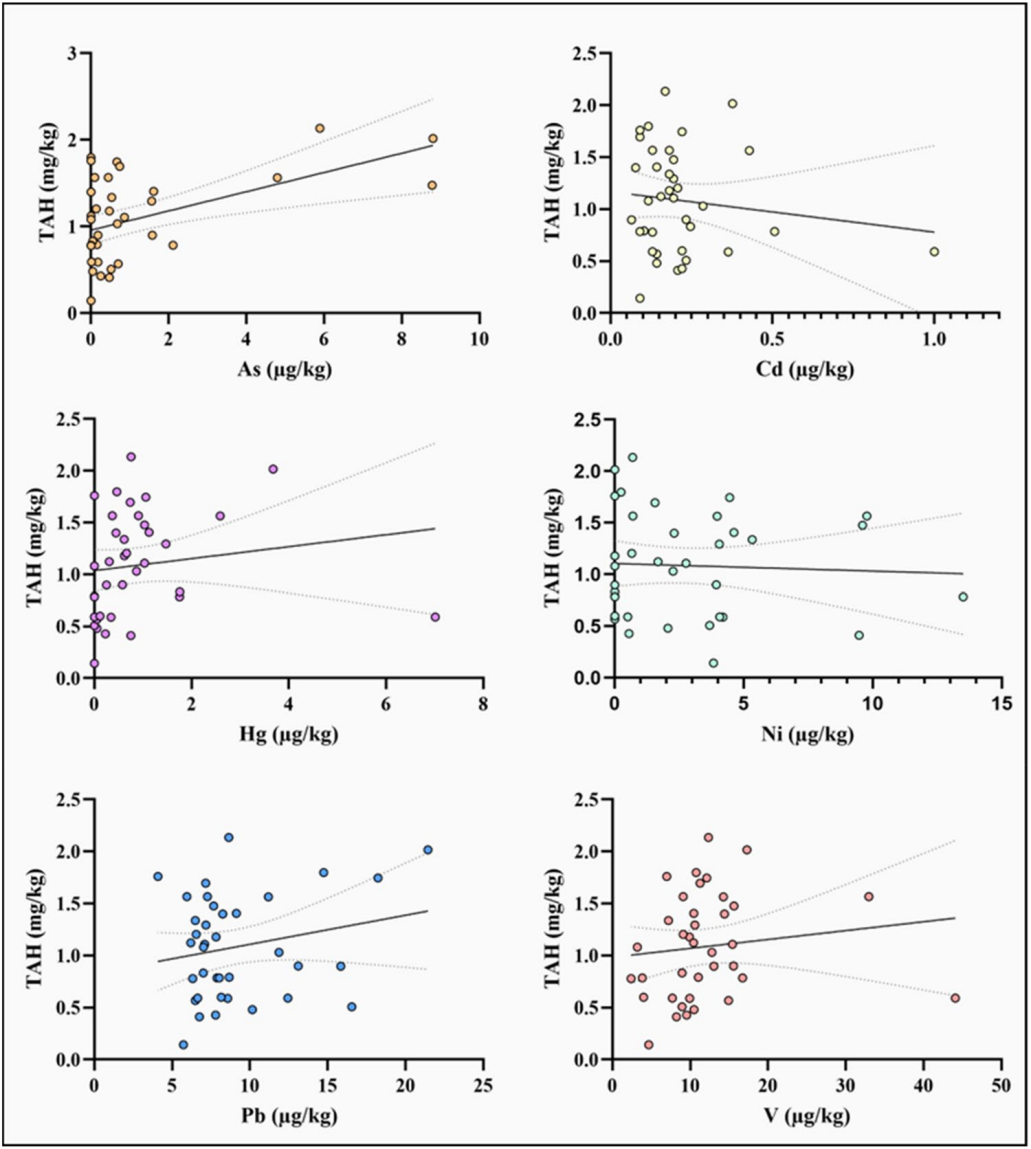
Scatter graphs between TAH and toxic elements in starry sturgeon samples from the Caspian Sea

Results of Spearman’s correlation analysis are shown in Table 2. Regarding the correlation between toxic elements and hydrocarbons, only Hg and V had a positive correlation with TAH. In this regard, the *r* values were 0.436 and 0.327 for Hg and V, respectively. In the case of As, its *p-value* was 0.056, a value that slightly exceeded the confidence level (*p* < 0.05), so we decided not to take it as a conclusive result. Figure 2 shows the scatter graphs between TAH and determined toxic elements, where different correlations can be seen. On the other hand, a high correlation between different toxic elements is proven, as shown in Table 2.

**Table 2:** Spearman’s correlation analysis between TAH and toxic elements in starry sturgeon

	Compound		TAH	As	Cd	Hg	Ni	Pb	V
Rho de Spearman	TAH	r	1	0.316	− 0.120	0.436**	0.035	0.119	0.327*
		Sig	-	0.056	0.478	0.007	0.838	0.481	0.048
	As	r		1	0.468**	0.623**	0.426**	0.351*	0.613**
		Sig		-	0.002	0.000	0.006	0.026	0.000
	Cd	r			1	0.593**	0.396*	0.343*	0.236
		Sig			-	0.000	0.011	0.030	0.143
	Hg	r				1	0.479**	0.339*	0.426**
		Sig				-	0.002	0.032	0.006
	Ni	r					1	0.088	0.341*
		Sig					-	0.590	0.031
	Pb	r						1	0.325*
		Sig						-	0.041
	V	r							1
		Sig							-

^*^The correlation is significant with a confidence level of 0.05 (bilateral)

^**^The correlation is significant with a confidence level of 0.01 (bilateral)

### Principal component analysis

**Fig. 3:**
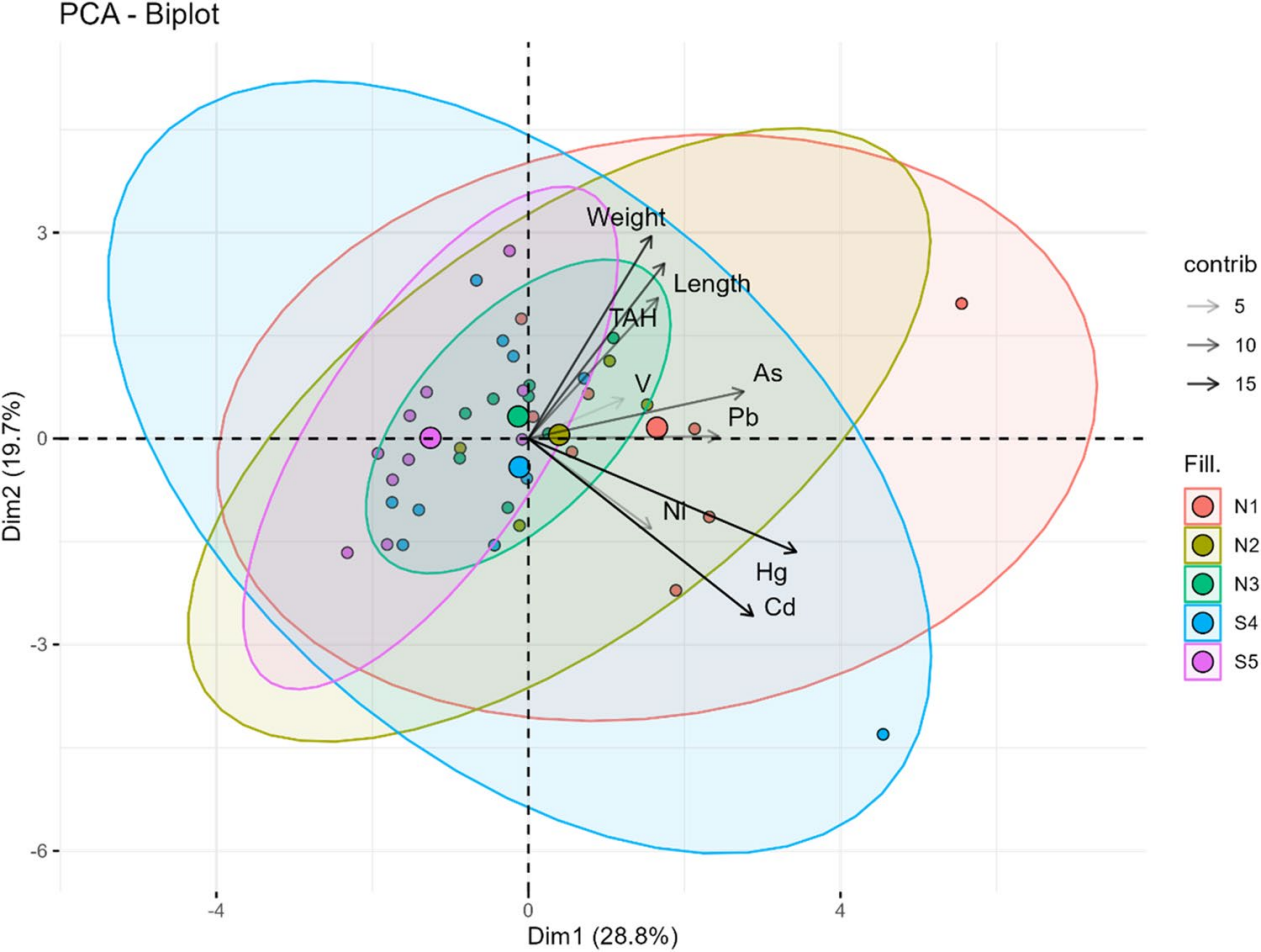
Spatial distribution in PC1 and PC2 of length, weight, TAH, and toxic elements determined in sturgeon in different locations in the Caspian Sea

In the PCA, four principal components (PCs) were extracted, whose eigenvalues exceeded the unity. The explained variance percentages for PC1, PC2, PC3, and PC4 were 28.8, 19.7, 14.3, and 13.7, respectively. With these four components were explained the 76.5% of the variance. Table 3 shows the loadings of height, weight, TAH, and toxic elements with each components. All loadings were positive for PC1 exceeding at all times 0.3, implying a strong involvement of all variables in this component. In the case of PC2, length, weight, TAH, As, and V had a positive loading, while the other variables were negatives or neutral such as Pb. Both components, PC1 and PC2, explained the 48.5% of the variance. Figure 3 shows the spatial distribution of each location with respect to these two components indicating, in addition, the contribution of each variable.

**Table 3:** Loadings between principal components of PCA and length, weight, TAH, and toxic elements determined in sturgeon

Variable	PC 1	PC 2	PC 3	PC 4
Length	0.4143844	0.606657537	− 0.4987555	0.309265161
Weight	0.3746787	0.701472591	− 0.4382613	− 0.001685339
TAH	0.3952502	0.487070566	0.5261645	− 0.308614944
As	0.6565125	0.162913141	0.5109190	− 0.085034259
Cd	0.6843315	− 0.614540093	− 0.3078367	− 0.024759511
Hg	0.8159013	− 0.393086210	− 0.2170338	− 0.152485259
Ni	0.3741725	− 0.312059554	0.1725340	0.607038169
Pb	0.5800106	0.007527265	0.0808575	− 0.406745431
V	0.2903158	0.137716950	0.3574629	− 0.481581355

## Discussion

The release of oil and oil-related products into the environment can destroy an ecosystem because these compounds are highly persistent, have a low biodegradability, are bioaccumulative, and possess a high biotoxicity (Chen et al. 2015; Lee et al. 2019; Ossai et al. 2020). The genetic reserves of sturgeon in the Caspian Sea form one of the largest natural resources in the world, and play a fundamental role in this sea’s ecosystem because, mainly, they are predators (Fazli et al. 2022). These fish live at the bottom of the sea and their feeding pattern, which is based on the ingestion of the benthic organisms found in its sediments, increases the risk of exposure to many pollutants, such as metals or crude oil hydrocarbons (Poorbagher et al. 2017). Moreover, these species are very long-lived, between 50 and 75 years (Doroshov 2019), so they are exposed to pollutants for a long time and can be good bioindicators of pollution. Particularly, we have observed in the PCA that variables such as weight and length (attributes related to the age of the sturgeon) are strongly associated with the presence of TAH in them. Therefore, detecting the concentrations of these contaminants in these species can provide highly valuable information for assessing the level of pollution to which these animals are exposed. That information could be used to establish area management actions, that will help to conserve this highly valued species, that is currently, under great threat.

Another key aspect is the matrix employed for TAH and metal determination. These sturgeons are “protected species” so sacrificing animals to determine concentrations in muscle and other organs is not justified. The use of bone samples from the marginal pectoral fin is a validated and minimally harmful method for sturgeons, and is mainly used to determine their age and growth (Baremore 2014; Bakhshalizadeh et al. 2021). In addition, several studies have reported that bone tissue is a good matrix to address the concentration of accumulative substances like toxic elements, since the concentrations obtained in this tissue are higher than those found in muscle tissue, and could be compared to those obtained in liver tissue (Edem et al. 2009; Adeosun et al. 2015). Future research could also consider the collection of blood samples through a non-invasive method to add information about the health of the sturgeons.

Regarding the presence of hydrocarbons in marine sediments, Readman et al. (2002) found that there is no contamination when their concentration is below 10 mg/kg, but that the sediments are polluted when their concentration is over 100 mg/kg. Shirneshan et al. (2017) reported concentrations of TAH in sediments in several areas in the south-western Caspian Sea, which varied from 19.75 to 996.23 mg/kg, which are considered relatively high. There are very few studies on the concentrations of aliphatic hydrocarbon in fish. De Mora et al. (2010) obtained concentrations of TAH in the muscles of hamour and tuna, which ranged between 0.56 and 9.9 mg/kg in the ROPME Sea Area, in which the oil industry is also strongly developed. The concentrations obtained in our study are in the range found by these researchers. On the other hand, the proximity to the Volga river, which carries a lot of waste, suggests that it could be responsible for the higher levels of TAH and some elements in northern areas. However, despite the high concentrations of TAH in Caspian Sea sediments, based on those obtained in our work, it is suggested that, surprisingly, they are not accumulated in excess in the sturgeons spine and that their concentration in other organs could be higher. According to Mohsenpour (2020) hydrocarbons accumulate in the liver and gall bladder after they enter the body orally. These authors also pointed out that one of the main adverse effects of oil hydrocarbons is that they alter and reduce fish fertility. In general, fish early life stages and juveniles across taxa may be more susceptible to the effects of oil due to their immaturity and developing physiologies (Takeshita et al. 2021). These adverse effects clearly can negatively affect repopulation programs of various sturgeon species.

Crude oils mainly consist of hydrocarbons but a small amount (generally under 1%) is composed of inorganic compounds, including metals or metalloids (Shahat et al. 2018). V is usually the most abundant trace metal in oil samples, and its concentration can reach up to 1500 mg/kg, although in some crude oils there is less than 0.1 mg/kg (Amorim et al. 2007). On the other hand, Ni concentrations may reach 340 mg/kg in crude oil. Ni/V ratios have been used to classify those in crude oils (Barwise 1990). In the Caspian Sea, V can be trapped in the oil production process, while fuel is burned, although generally its concentration in the water is insignificant and it does not affect its quality (Tasmagambetova et al. 2019). Concentrations of Ni found in sturgeons generally agree with the values reported previously in different tissues of other species (0.1–8.00 mg/kg) in the Caspian Sea (Sheikhzadeh and Hamidian 2021). This suggest that the concentrations of TAH, the most abundant compounds in crude oil, are probably closely related to the concentrations of Ni and V since these two metals should be the most abundant. In the present work, the Spearman correlation, which focuses on evaluating the relationship between pairs of variables, for example, TAH and V, and PCA, which identifies general patterns in a multidimensional dataset, has been used. Using both approaches provides a more comprehensive understanding of the relationships between TAH and toxic elements in the study. In the Spearman correlation, positive correlations have been found between Hg and V with respect to TAH. Meanwhile, in the PCA, all elements have shown a strong correlation with TAH in PC1, particularly Hg, and only As and V in PC2. This could imply that all the elements share some more or less close environmental relationship, with As, Hg, and V being the ones most related to petroleum. Apart from the harmful effects of oil itself, the adverse effects of As on fish health include various mechanisms of acute and chronic toxicity, ranging from enzymatic, genetic, and immune system failures, which alter normal biological functions, resulting in the direct initiation of diseases or, at least, in the body’s predisposition to them (Kumari et al. 2016). Regarding this concern, Acolas et al. (2020) determined As concentrations of less than 0.25 µg/mL in European sturgeon (*Acipenser sturio*) in the Gironde estuary in France. Although it is true that they used a different biological matrix, such as blood, these are much lower concentrations than those determined in any area of this study. On the other hand, Hg extreme toxicity, long-distance migration, and bioaccumulation are a serious problem for ecosystems. At the moment, several methods are being applied to Hg capture since it is a major challenge in petroleum and natural gas processing (Warrag et al. 2018; Huo et al. 2019). Overall, oil pollution, already highly concerning on its own, carries with it the pollution of toxic elements that further worsens this environmental issue in the Caspian Sea.

## Conclusions

Monitoring contaminants directly on protected species, in addition to the environment where they live, can add information on the bioaccumulation of pollutants, such as hydrocarbons. It should be noted that monitoring these species requires non-lethal methods, which can pose challenges to the study. In this case, the presence of aliphatic hydrocarbons from oil in all samples of sturgeon spines has been evidenced in the southern and northern Caspian Sea, particularly with higher concentrations in the northern areas. Furthermore, to a greater or lesser extent, some toxic elements, mainly Hg and As, have been linked to TAH, which aggravates this environmental problem due to their toxicity. So, proposing new studies on sturgeons themselves using other non-lethal methods or evaluating their environment is mandatory to recover valuable and unique species such as sturgeons. Finally, it should be emphasized that restrictions must be taken against pollution in the Caspian Sea.

## Data Availability

The data that support the findings of this study are available from the corresponding author.
